# Emerging story of gut dysbiosis in spondyloarthropathy: From gastrointestinal inflammation to spondyloarthritis

**DOI:** 10.3389/fcimb.2022.973563

**Published:** 2022-08-22

**Authors:** Xing Lyu, Jieli Chen, Xingjie Gao, Jie Yang

**Affiliations:** ^1^ Department of Rheumatology and Immunology, Tianjin Medical University General Hospital, Tianjin, China; ^2^ Discipline Construction Office, Tianjin Medical University, Tianjin, China; ^3^ Department of Biochemistry and Molecular Biology, Department of Immunology, Key Laboratory of Immune Microenvironment and Disease (Ministry of Education), Key Laboratory of Cellular and Molecular Immunology in Tianjin, Excellent Talent Project, The Province and Ministry Co-sponsored Collaborative Innovation Center for Medical Epigenetics, School of Basic Medical Science, Tianjin Medical University, Tianjin, China

**Keywords:** spondyloarthritis, inflammation, gut-joint axis, probiotics, gut dysbiosis

## Abstract

As a set of inflammatory disorders, spondyloarthritis (SpA) exhibits distinct pathophysiological, clinical, radiological, and genetic characteristics. Due to the extra-articular features of this disorder, early recognition is crucial to limiting disability and improving outcomes. Gut dysbiosis has been linked to SpA development as evidence grows. A pathogenic SpA process is likely to occur when a mucosal immune system interacts with abnormal local microbiota, with subsequent joint involvement. It is largely unknown, however, how microbiota alterations predate the onset of SpA within the “gut-joint axis”. New microbiome therapies, such as probiotics, are used as an adjuvant therapy in the treatment of SpA, suggesting that the modulation of intestinal microbiota and/or intestinal barrier function may contribute to the prevention of SpA. In this review, we highlight the mechanisms of SpA by which the gut microbiota impacts gut inflammation and triggers the activation of immune responses. Additionally, we analyze the regulatory role of therapeutic SpA medication in the gut microbiota and the potential application of probiotics as adjunctive therapy for SpA.

## Introduction

Spondyloarthritis (SpA) is a family of clinical disorders with some featured manifestations, etiopathological characteristics and genetic factors (Moz et al., 2017; Duba and Mathew, 2018; Terenzi et al., 2018). SpA primarily features sacroiliitis, namely, axial SpA (axSpA), and the peripheral joint can also be involved (pSpA) (Sieper et al., 2009; Rudwaleit et al., 2011; Stolwijk et al., 2012; Dubreuil and Deodhar, 2017; van der Heijde et al., 2017). Back pain is a major symptom of axSpA, and exercise can improve pain and stiffness (Rudwaleit and Sieper, 2012; Bidad et al., 2017). Additionally, there are other musculoskeletal manifestations (e.g., arthritis, enthesitis, dactylitis, etc.) and extra-articular manifestations (e.g., psoriasis, anterior uveitis, etc.) for axSpA (Duba and Mathew, 2018; Magrey et al., 2020). In recent years, the term “axSpA” has been used more commonly to cover this group of SpA diseases (Sieper and Poddubnyy, 2017; Ritchlin and Adamopoulos, 2021; Robinson et al., 2021; Danve and Deodhar, 2022).

Based on the sacroiliac joint radiological results, axSpA can be classified further into radiologically positive axSpA with definite imaging damage (r-axSpA) and radiologically negative axSpA, namely, non-radiographic axSpA (nr-axSpA) (Sieper and Poddubnyy, 2017; Ritchlin and Adamopoulos, 2021; Robinson et al., 2021; Danve and Deodhar, 2022). There are some patients with nr-axSpA who progress to r-axSpA (Protopopov and Poddubnyy, 2018). Ankylosing spondylitis (AS) is the best studied subtype of axSpA at the later stage (Dougados and Baeten, 2011; Sieper and Poddubnyy, 2017). Enteropathic arthritis (EPA), reactive arthritis (ReA), and psoriatic arthritis (PsA) are the common types of pSpA (Wilson and Folzenlogen, 2012; Taams et al., 2018). New imaging modalities, biomarkers, and genetic data may be available to update the classification criteria of SpA (Sieper et al., 2009; Rudwaleit et al., 2011; Dubreuil and Deodhar, 2017; van der Heijde et al., 2017). Emerging evidence supports the idea that SpA and inflammatory bowel disease (IBD) share similarities in their genetic predisposition and pathogenesis (Olivieri et al., 2014; Karreman et al., 2017; Fragoulis et al., 2019; Qaiyum et al., 2021). Thus, IBD-associated SpA was a concern in this study.

Based on the most recent research on the relationship between SpA and intestinal inflammation, we summarize the evidence that supports intestinal dysbiosis in SpA. Specifically, the mechanisms by which dysbiosis contributes to subclinical intestinal inflammation and immune response activation to SpA will help us to understand the idea of the “gut-joint axis”. To this end, we outline the effects of current SpA medication treatment options on the gut microbiota with a focus on research findings in the field of microbiota-targeted precision therapy. Lastly, some thoughts were provided on the underlying mechanisms and modulation of the intestinal microbiota as potential new treatment approaches to SpA.

## Intestinal microbial dysbiosis in SpA

The human microbiota contains a plethora of parasitic or symbiotic microorganisms, such as bacteria, viruses, and fungi (Miller et al., 2021). The intestine is crucial for maintaining homeostasis between the microbiota and the host, which involves the effective coordination of different immune cells (Tiffany and Bäumler, 2019). The number of cells in the typical flora of the human colon has been estimated to reach 10^12^ colony forming units (CFU) (Sekirov et al., 2010). The intestinal flora primarily consist of *Bacteroidetes*, *Firmicutes*, *Actinobacteria*, and *Proteobacteria* (Honda and Littman, 2012). The gut microbiota is associated with human metabolic and physiological activities, including balancing the immune response and regulating intestinal endocrine function and amino acid metabolism (Honda and Littman, 2012; Lynch and Pedersen, 2016; Dupont et al., 2020). Dysbiosis means the occurrence of a microbiome imbalance when environmental factors or host-related factors alter the composition and function of microbial communities, which is related to a series of clinical disorders (Brandl and Schnabl, 2015; Tiffany and Bäumler, 2019).

Emerging publications report the relationship between the gut microbiota and SpA. We summarized the recent findings on the changes in the gut microbiota under SpA in **Table 1**. There is no consensus on which bacterial species are linked to the development of SpA. Several candidate microbes were identified as potential drivers of gut inflammation in experimental SpA induced by HLAB27 (Gill et al., 2019). Prevotella and Blautia (Lewis rats) and Akkermansia muciniphila, rc4-4, Lachnospira, and Lachnospiraceae (Fischer rats) were found to be closely related to the dysregulated inflammatory pathways (Gill et al., 2019). The emergence of joint damage resembling Reiter’s syndrome in a ship’s crew sick with bacterial dysentery prompted researchers to investigate the link between the intestine and arthritis (Noer, 1966). Mounting evidence supports the functional links of SpA and gastrointestinal inflammation (Rizzo et al., 2018), which are closely related to intestinal microbial dysbiosis (Ni et al., 2017). Animal experiments have revealed that transgenic rats harboring HLA-B27 displayed clinical symptoms comparable to human SpA but without further development under sterile conditions, which indicates that intestinal microbes are essential for the onset of SpA (Taurog et al., 1994).

**Table 1:** Recent findings on changes in the SpA gut microbiota.

	Study Subjects	Key findings	Ecological changes in the flora	Strains with increased abundance		Strains with reduced abundance	
1	Microbial profiles for terminal ileum biopsy specimens obtained from patients with recent-onset tumor necrosis factor antagonist-naive AS (Costello et al., 2015)	Microbial communities in AS differ significantly from those in healthy control subjects, driven by a higher abundance of 5 families of bacteria	The microbial composition was demonstrated to correlate with disease status	*Lachnospiraceae, Ruminococcaceae, Rikenellaceae, Porphyromonadaceae*, *Bacteroidaceae*		*Veillonellaceae*, *Prevotellaceae*	
2	Stool specimens from 150 AS patients (Klingberg et al., 2019)	There is a distinct fecal microbiota profile, which is associated with the fecal calprotectin levels.	87% of patients with ecological disorders	*Proteobacteria*, *Enterobacteriaceae*, *Bacilli*, *Streptococcus* spp.*, Actinobacteria*		*Bacteroides, Lachnospiraceae*	
3	Chinese AS patient cohort (Wen et al., 2017)	Reduced abundance of melanin-producing *Prevotella*, *Prevotella* spp. and *Anaphyllobacter* spp.	Ecological disorders			*Bacillus* spp., *Prevotella, melanogaster, Prevotella* spp.	
4	Stool samples from 22 patients with AS (Li et al., 2019)	Increased abundance of *Bacillus variegatus* and reduced *Bacillus mimicus*	Lower biodiversity ratios; significant reduction in the diversity of intestinal fungi	*Ascomycota*, *Cysticercus*		*Basidiomycota*, *Stretchers*	
5	Stool samples from two AS cohorts (Breban et al., 2017)	*Ruminal cocci* may be a potential marker of disease activity	A unique ecological disorder	align="left"> *Rumenococcus*			
6	27 patients with SpA (Tito et al., 2017)	*Dialister* may be a potential microbial marker of disease activity	Significant differences in the microbiological composition of the gut in patients with microscopic intestinal inflammation	*Dialister*			
7	Macrogenome sequencing of stool samples from patients with IBD (Hall et al., 2017)	Significant increase in the abundance of parthenogenic anaerobic bacteria tolerant to oxidative stress; dramatic but transient rumen cocci blooms coinciding with increased disease activity	Low diversity	*Rumenococcus*, Parthenogenic anaerobic bacteria			
8	A total of 174 mucus samples from 43 UC and 26 CD patients (Nishino et al., 2018)	Significant increase in the Metaplasma phylum and significant decrease in the phylum *Firmicutes* and *Bacteroidetes*; CD and UC have different microbial community structures associated with mucous membranes.	Significant reduction in alpha diversity	Phylum *Metaplasma*		Phylum *Firmicutes*, *Bacteroidetes*	
9	Stool analysis of patients with IBD (Alam et al., 2020)	The changed bacterial groups are those that do not co-exist well with the common intestinal commensal bacteria	Low microbiome diversity	*Coriobacteriaceae, Prevotellaceae, Burkholderiaceae, Veillonellaceae, Streptococcaceae, Pseudomonadaceae, Acidaminococcaceae*			
10	Recent-onset, DMARD-naive PsA (Scher et al., 2015)	Low relative abundance of *Akkermansia* and *Ruminoccocus* hb as a characteristic of the PsA gut microbiota	Reduced diversity of the gut microbiota due to the low relative abundance of several taxa.			*Akkermansia*, *Ruminoccocus*	
11	52 psoriasis patients (Codoner et al., 2018)	Type 2 patients have a higher frequency of bacterial translocation and more frequent inflammatory states		*Faecalibacterium*		*Bacteriodes*	

## Mechanisms of microbiota-derived intestinal inflammation in SpA

Microbial dysbiosis of gut commensals has been implicated in SpA and is highly related to gut inflammation (Gill et al., 2015). Patients with SpA frequently suffer from IBD (Fragoulis et al., 2019), and there are similarities in the aberrations of the gut microbiota under IBD and AS (Klingberg et al., 2019). The mechanism underlying how intestinal inflammation causes immune damage to peripheral organs is currently inconclusive. Three points are under consideration. (1) There may be no inherent relationship, but rather a simple overlap of clinical characteristics, between axSpA with subclinical intestinal inflammation and IBD with sacroiliitis. (2) A-specific factor causes SpA to experience both intestinal and joint inflammation in parallel. Genetic, immune, and environmental factors all have a direct impact on the immune system, which in turn induces the clinical immune feature of SpA. This may partly explain why some individual SpA cases have only joint immune changes but no intestinal symptoms. (3) The “gut-joint axis” hypothesis, which is currently very popular, indicates possible links between intestinal and joint pathology. Genetic, immune, and environmental factors first affect the gut and induce dysbiosis in the microbiota through a series of regulatory mechanisms (e.g., arthritic peptide recognition, clonal expansion, etc., and ultimately lead to the inflammation of other sites, such as joints (Gracey et al., 2020; Zaiss et al., 2021).

Mounting evidence supports the functional link between the gut dysbiosis-related immune system response and SpA. Th17 cells are vital in host defense against external microbes and fungi, and IL-23 signaling is required for the maturation and stability of the pro-inflammatory Th17 phenotype (Schinocca et al., 2021). The IL-23/IL-17 axis and associated cytokines have been implicated in the etiology of SpA (Tsukazaki and Kaito, 2020; Lukasik et al., 2021). In addition to Th17 cells, some innate-like T-cell subsets that express the IL-23 receptor, such as mucosa-associated invariant T (MAIT) cells, T cells, invariant natural killer T cells (iNKT), and type 3 innate lymphocytes (ILC3), boost the type 3 immune response and play a role in the pathogenesis of SpA (Sherlock et al., 2012; Mauro et al., 2019). For instance, upon stimulation with bacterial products, ILC3s can induce an inflammatory response through the production of IL-17 and link the gut microbiota and local/systemic immunity (Annunziato et al., 2015; Asquith et al., 2019). Other immune cells, including mast cells and macrophages, can further amplify the inflammatory effect by secreting cytokines such as IL-23, IL-17, IL-1, IL-22, and tumor necrosis factor (TNF)-α (Annunziato et al., 2015). MAIT cells are capable of producing pro-inflammatory cytokines, such as IL-17, IL-22, TNF, or interferon (IFN)-γ (Gracey et al., 2016; Toussirot and Saas, 2018; Li et al., 2022). Taken together, these findings show that various immune cell-mediated IL-23/IL-17 axes contribute to the migration of blood vessels.

**Figure 1:**
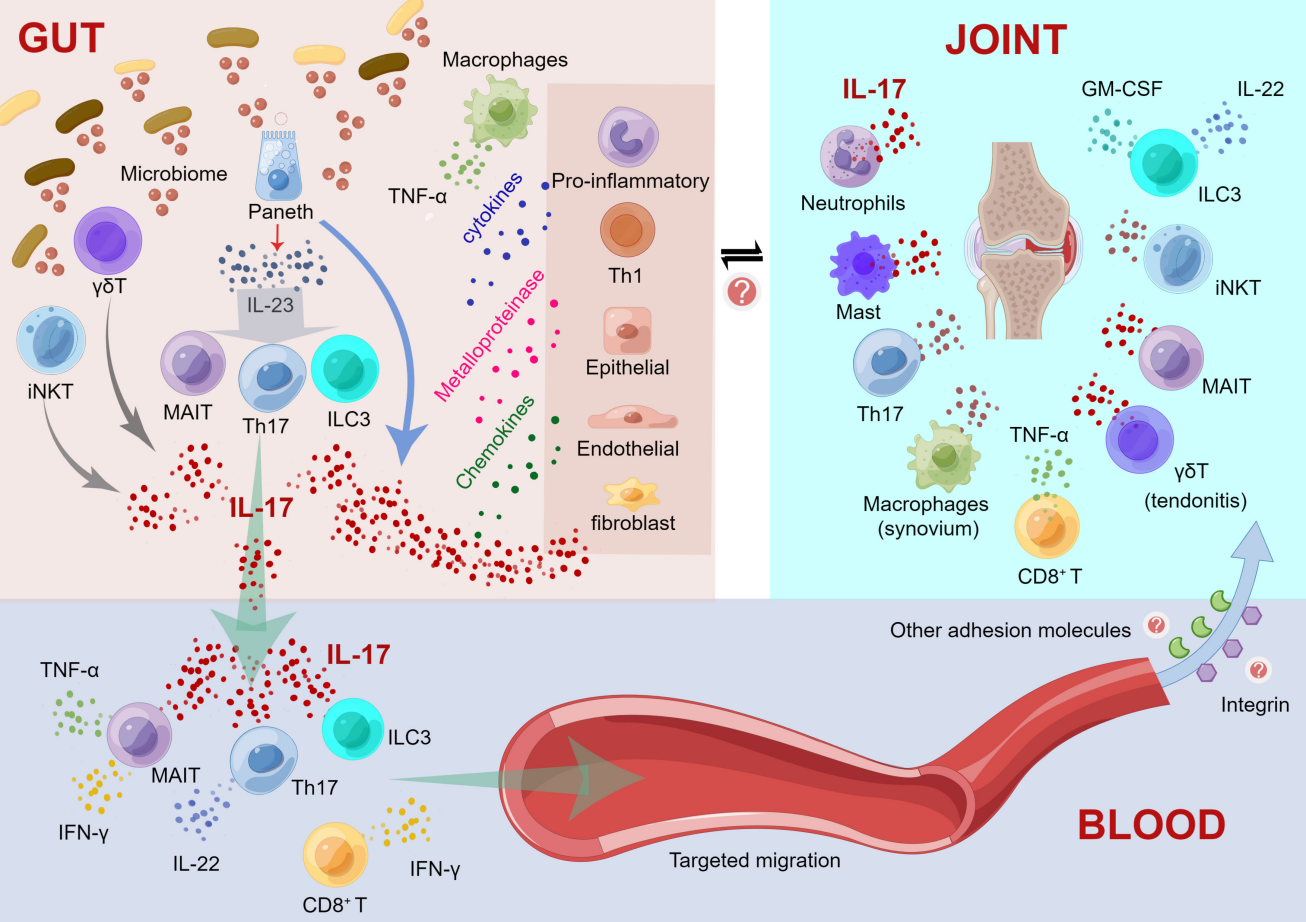
Mechanistic model regarding the intestinal inflammation driving immune damage in peripheral joints. **(1) Immunological changes in the gut**. a) Activated Paneth cells produce IL-23 and IL-17 after recognizing the altered microbiome; IL-23 causes the differentiation of Th17, ILC3 and MAIT cells; and these activated cells foster the production of elevated levels of IL-17. IL-17 acts as an inflammatory mediator to stimulate the production of cytokines by other proinflammatory cells and Th1 cells and promotes the production of metalloproteinases and chemokines by macrophages, epithelial cells, endothelial cells, and fibroblasts, thereby triggering and maintaining inflammation. b) γδ T and iNKT cells can recognize microbial antigens and release IL-17. c) Simultaneously, macrophage recruitment promoted the secretion of TNF-α. **(2) Cytokine cascades initiated in blood vessels.** a) IL-17, Th17 cells, ILC3 and MAIT cells can migrate through the intestinal mucosal barrier to the blood, and these cells may transfer inflammation to the joints. b) Th17, γδT, ILC3 cells and MAIT cells are induced to produce IL-17 in the bloodstream; Th17 cells also produce IL-22. c) MAIT cells also contribute to the production of TNF-α and IFN-γ. d) CD8^+^ T cells produce IFN-γ. **(3) Targeting migration and immune response of extraintestinal joint sites**. a) Activated cytokines migrate to inflammatory sites, such as the axial/peripheral joints, for immune interference. b) Some adhesion molecules, such as integrins, may contribute to the target migration of these immune cells circulating in the blood to peripheral joints. c) In peripheral joints, neutrophils, mast cells, Th17 cells, CD8^+^ T cells, MAIT cells and iNKT cells induce IL-17 production. ILC3 cells produce IL-22 and GM-CSF, whereas IL-17 is only produced in axial joints. d) γδT cells promote tendonitis through elevated IL-17. e) Macrophages induce TNF-α production in the synovium. This figure was drawn by Figdraw.

Here, we propose an IL-23/IL-17 axis-based mechanistic model regarding intestinal inflammation driving immune damage in peripheral joints. As shown in **Figure 1**, our model includes three parts, namely, “immunological changes in the gut”, “cytokine cascades initiated in blood vessels”, and “targeting migration and immune response of extraintestinal joint sites”. Detailed information is shown in the legend of **Figure 1**. Accumulating evidence suggests that impaired intestinal barrier structure and function, as characterized by altered tight junction protein density and high intestinal permeability, may increase the entry of bacterial and/or microbiota-derived inflammatory components into the circulatory system (Vaile et al., 1999; Gill et al., 2015; Ciccia et al., 2017). For instance, as a modulator of high intestinal permeability, zonulin protein is highly expressed and implicated in damage to the intestinal mucosal barrier and gut vascular barrier (Ciccia et al., 2017). Fluctuations in the amount, variety, and function of the core gut microbiome can cause increased gut permeability (Brandl and Schnabl, 2015). Herein, IL-23/IL-17 axis-mediated gut bacteria and metabolites influence gut immune mechanisms and disrupt the gut barrier integrity. The “aberrant cell trafficking hypothesis (recirculation)” mimics the recruitment of mucosal-derived cells to joints, in which T cells and macrophages in the gut activate and then recirculate to the joints (Qaiyum et al., 2021). Evidence on adhesion molecules (e.g., integrin, etc.) that determine the homing pattern of circulating lymphocytes may be involved in the mechanism and process of immune-competent cell migration from the intestinal mucosa. For instance, MAIT cells have been isolated from the synovial fluid of AS patients (Gracey et al., 2016). Integrin-expressing T cells proliferate in the inflamed joints of AS patients, and α4β7 integrin promotes T lymphocyte migration from the gut to the synovium (Qaiyum et al., 2019). Thus, cytokine cascades of IL-17 are initiated in blood vessels and migrate to the extraintestinal axial/peripheral joint sites for immune interference, probably through an adhesion molecular-involved mechanism.

## SpA therapeutic medication and gut microbiome

Currently, non-steroidal anti-inflammatory drugs (NSAIDs) are the first choice for pharmacological treatment of axSpA, according to the guideline panel (Sieper and Poddubnyy, 2017; Ritchlin and Adamopoulos, 2021; Robinson et al., 2021; Danve and Deodhar, 2022). Disease-modifying anti-rheumatic drugs (DMARDs) have some effect on axSpA patients with peripheral arthritis, but their efficacy in most SpA patients remains debatable (Ward et al., 2019). DMARDs can be further divided into several categories, including traditional DMARDs or conventional synthetic DMARDs (csDMARDs), biologic DMARDs (bDMARDs), and targeted synthetic DMARDs (tsDMARDs) (Buer, 2015; Smolen et al., 2020). bDMARDs and tsDMARDs are recommended for adults with active AS despite treatment with NSAIDs (Ward et al., 2019). Considering the close links between SpA and the gut microbiota (**Table 1**), we reviewed the potential association between different SpA therapeutic medications and the gut microbiome.

### NSAIDs

For SpA patients, NSAIDs can effectively alleviate the symptoms of SpA, such as morning stiffness and joint pain (Queiro-Silva et al., 2021). Even so, different NSAID treatments can cause a distinct alteration in the bacterial composition, which contributes to intestinal damage during NSAID treatment (Rogers and Aronoff, 2016; Otani et al., 2017). A reduction in mouse NSAID-induced enteropathy can be achieved by inhibiting bacterial β-glucuronidase enzyme activity (Saitta et al., 2014).

### csDMARDs

Even though csDMARDs [e.g., methotrexate (MTX), sulfasalazine (SSZ), hydroxychloroquine (HCQ), etc.] are not the first choice for axSpA, the subtypes of peripheral joint manifestations for PsA and EPA involve csDMARDs (Mease, 2012; Chimenti et al., 2019; Scher et al., 2020; Jacobs et al., 2021). The treatment efficacy of the MTX and SSZ combination was observed for active axSpA patients in a prospective cohort study (Ganapati et al., 2021). Mounting evidence supports the functional links of csDMARDs and gut microbiota. For instance, the gut microbiome is essential for the treatment effect and prognostic evaluation of MTX for patients with rheumatoid arthritis (RA) (Scher et al., 2020; Artacho et al., 2021). Restored gut dysbiosis symptoms were observed in a rat model of experimental colitis after treatment with SSZ (Zheng et al., 2017). Following treatment with a short-term high dose of HCQ, female C57BL/6J mice showed altered gut microbiota rather than immunological responses (Pan et al., 2021). However, there are few studies on the effect of csDMARDs on the intestinal flora of SpA, which merits further investigation.

### bDMARDs

Tumor necrosis factor inhibitors (TNFi) and interleukin-17 inhibitors (IL-17i), two main types of bDMARDs, can be considered for patients who have failed or are intolerant to NSAID therapy (van der Heijde et al., 2017). TNF-α antagonists have specific therapeutic effects on SpA patients (Navarro-Compán et al., 2021) and exhibit considerable microbiota recovery (Chen et al., 2021; Ditto et al., 2021). Furthermore, there is a cross-influence between TNFi treatment and intestinal microbiota. For instance, SpA patients may respond to anti-TNF-α treatment more effectively if they exhibit a specific fecal microbiota signature (Bazin et al., 2018). Some microbes can serve as indicators of the therapeutic responsiveness of TNFi treatment for AS patients (Zhang et al., 2020). The lowering of bacterial arthritic peptides has been demonstrated to contribute to the improvement of the gut microbiome following TNFi treatment (Yin et al., 2020). Recent studies targeting IL-17 have provided new ideas for treating refractory SpA that has not responded to previous treatments (Gladman et al., 2019; van der Heijde et al., 2020; Schett et al., 2021). The IL-17 axis has a great deal of promise in terms of enhancing SpA therapy options (Smith and Colbert, 2014), and the potential role of gut dysbiosis can be considered.

### tsDMARDs

tsDMARDs, such as Janus kinase (JAK) inhibitors, have begun to be used in clinical trials with promising outcomes (Gladman et al., 2017; Toussirot, 2022). There have been limited studies of longitudinal changes in the gut microbiome with tsDMARD treatment in SpA. JAK inhibition has been found to have an indirect effect on the production of critical cytokines implicated in the pathogenesis of SpA as well as the triggering and maintenance of immunological responses (Veale et al., 2019; Ritchlin and Adamopoulos, 2021).

## New microbiome therapies such as probiotics

Probiotics have emerged as a new topic of SpA study as research into the involvement of gut microorganisms in the pathophysiology of the disease continues to intensify. Probiotics are described as living microorganisms that provide health advantages to the host when given in sufficient amounts (Hill et al., 2014). *Lactobacillus*, *Bifidobacterium*, and yeast are the most prevalent probiotics (Ashraf and Shah, 2014; Cunningham et al., 2021). Probiotics can be made up of a single strain, a mixture of strains, or a combination of both.

### Mechanism of action of probiotics

Probiotics have been researched *in vivo*, *in vitro*, and in animal models for their anti-inflammatory properties. Probiotics have been shown to improve the microbiota by modifying the gut environment, suppressing harmful bacterial growth, and preventing further immune system damage caused by inflammatory diseases (Shamoon et al., 2019). The mechanism of action of probiotics is usually strain-specific, interacting with the host and microbiome primarily through molecular effectors present on cell structures or secreted as metabolites (Cunningham et al., 2021). The basic mechanisms of action include promoting the growth of beneficial microorganisms in the intestinal microbiota, influencing immunological function, strengthening the intestinal barrier, competing with harmful microbes in the gut, and producing organic acids and antimicrobial compounds (Plaza-Diaz et al., 2019).

The immune response can be modulated by influencing cells involved in innate and adaptive immunity. Epithelial cells, dendritic cells (DCs), natural killer cells (NKs), macrophages, and lymphocytes are all affected by probiotics through the Toll-like activation of signaling pathways that regulate cell proliferation and cytokine production (Bermudez-Brito et al., 2012). The ability of probiotics to modulate the cytokine profile of DCs is strain-specific (Borchers et al., 2009; Cristofori et al., 2021)Furthermore, some probiotics can mediate the differentiation from B cells to IgA-producing plasma cells, and secretory IgA protects against infections by restricting bacterial attachment to the epithelium and inhibiting the penetration of host tissue (Liu et al., 2018).

**Figure 2:**
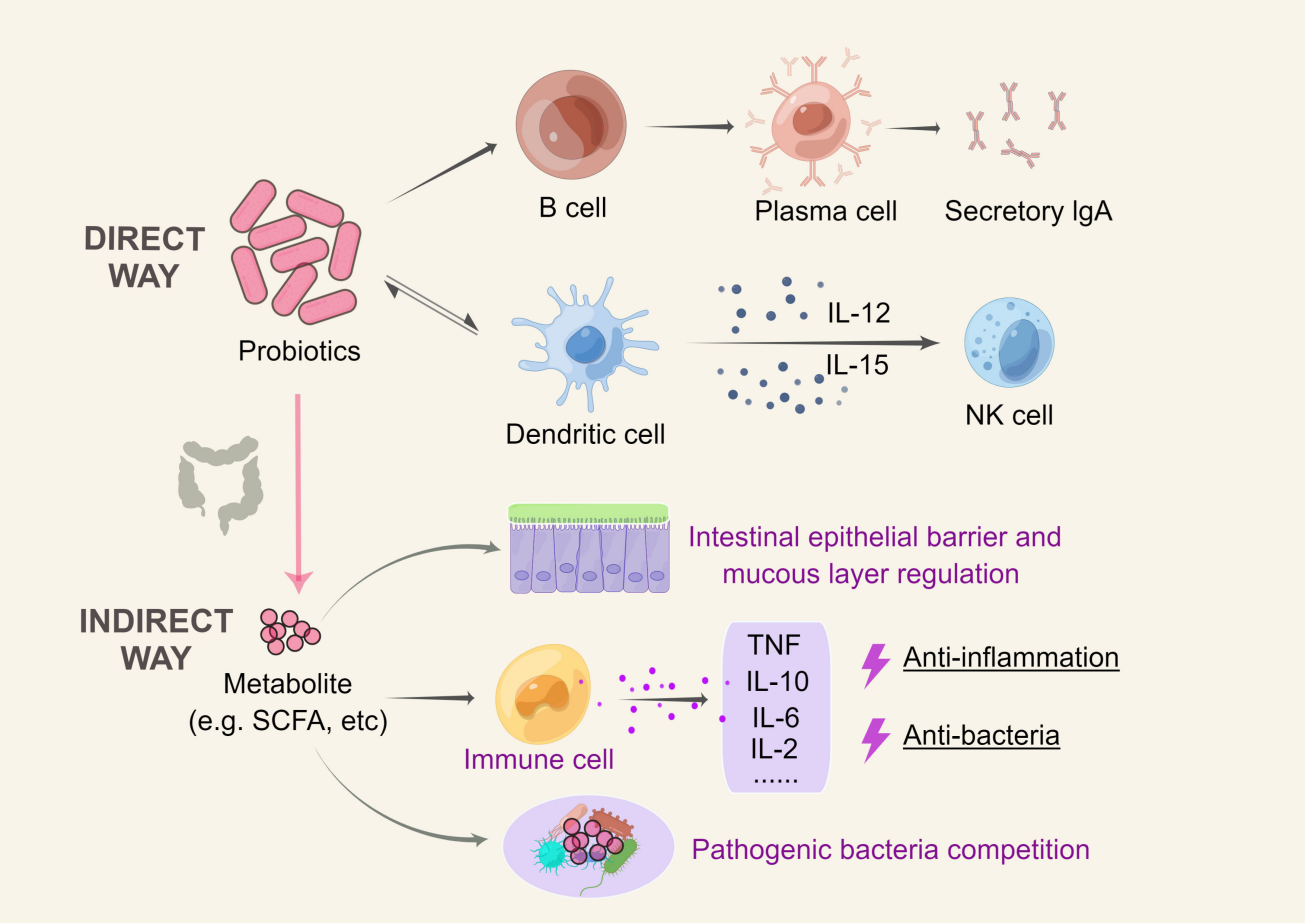
Mechanism of action of probiotics. **Direct mechanism:** probiotics can activate sentinel cells through the Toll-like activation of signaling pathways; DCs can drive NK cell activation by secreting cytokines such as IL-12 and IL-15, and probiotics can impact this pathway; probiotics can cause B cells to differentiate into IgA-producing plasma cells; and probiotics also interact with antigen-presenting cells to influence the reduction of proinflammatory cytokines, thereby triggering an adaptive response. **Indirect mechanism:** Through the manipulation of the gut epithelial barrier and mucus layer properties, probiotic release of antimicrobial compounds, and management of competition with pathogenic bacteria, specific probiotic metabolites may exert anti-inflammatory and antibacterial effects. This figure was drawn by Figdraw.

Probiotics can also engage with the host immune system indirectly. Through the manipulation of the gut epithelial barrier and mucus layer properties, the release of antimicrobial compounds, and the management of competition with pathogenic bacteria, specific probiotic metabolites may exert anti-inflammatory and antibacterial effects. Immune responses and systemic inflammation are also influenced by probiotic-driven metabolites such as short-chain fatty acids (SCFAs), which modulate immune cell activity (Oliviero and Spinella, 2020). This characteristic may contribute to the correction of intestinal wall hyperpermeability in the “gut-joint axis”. The major mechanism of probiotic action is illustrated in **Figure 2**.

### Clinical studies of probiotics as adjunctive therapy in SpA

Probiotics can theoretically be used to manipulate the microbiome as a promising adjunct therapy for SpA. As mentioned above, there are multiple extraintestinal manifestations of IBD, of which arthritis is one of the most common and is defined as EPA, an important member of the SpA disease spectrum. In this study, we provide a summary of current clinical trials using probiotics for IBD in **Table 2**. The number of controlled studies on probiotics for SpA is small, and the results of the few studies that have been conducted are not encouraging (Sanchez et al., 2022). Sanges et al. reported that a probiotic mixture containing *Lactobacillus acidophilus* and *Lactobacillus salivarius* is able to reduce arthritis disease scores in SpA patients with quiescent colitis, which may help in the management of SpA in patients with ulcerative colitis (Sanges et al., 2009). The findings of Lowe et al. suggest that combining *Bifidobacterium* and *Lactobacillus* preparations may have benefits in terms of pain relief, lower C-reactive protein (CRP), and improved quality of life. However, compared to RA patients, SpA patients did not benefit as much from CRP reduction (Lowe et al., 2020).

**Table 2:** Summary of population-based clinical trials on probiotics for IBD.

Object	Probiotic strains	Study results	Reference
IBD in remission or with mild symptoms	*Lactobacillus plantarum, Lactobacillus rhamnosus, Lactobacillus acidophilus, Enterococcus faecalis*	No difference in clinical symptoms after treatment	(Bjarnason et al., 2019)
CD	*Lactobacillus* GG	Not effective in preventing relapse	(Bousvaros et al., 2005)
UC	*E. coli* Nissle1917	Appears to be expected to maintain a period of remission	(Kruis et al., 2004)
UC	*Lactobacillus delbrueckii* *Lactobacillus fermentum*	Reduces NF-kB regulation and further reduces IL-6 and TNF- α levels	(Hegazy and El-Bedewy, 2010)
UC	*Bifidobacterium infantis*	CRP and TNF- α levels were reduced, but there was no significant effect on the course of UC; induces NF-kB regulation and further reduces IL-6 and TNF-α levels	(Groeger et al., 2013)
UC	*Bifidobacterium* Short *Bifidum perfringens* strain	Better endoscopic scores obtained, but no significant effect on UC	(Ishikawa et al., 2011)
UC	*Bifidobacterium breve*, *Lactobacillus acidophilus*	No significant improvement observed	(Matsuoka et al., 2018)
UC	*Lactobacillus acidophilus*, *Bifidobacterium animalis subsp. lactis*	Remission in 25% of the patient group, 8% of the placebo group. No significant difference	(Wildt et al., 2011)
Mild to moderate UC	*Bifidobacterium longum*	Reduced disease activity index, reduced rectal bleeding and clinical remission	(Tamaki et al., 2016)
UC	*Lactobacillus salivarius*, *Lactobacillus acidophilus*, *Bifidobacterium bifidum*	Have a positive effect	(Palumbo et al., 2016)
Mild to moderately active UC	VSL#3 (*Lactobacillus, Bifidobacterium*, *Streptococcus thermophilus*)	Significant improvements in rectal bleeding and stool frequency, mucosal appearance and overall assessment by the doctor	(Sood et al., 2009)
Mild to moderate UC that does not respond to conventional treatment	VSL#3	Remission/response rate of 77% with no adverse events.	(Bibiloni et al., 2005)

The efficacy of probiotics in SpA is currently uncertain. There are several causes for this outcome. First, the quality of all current clinical data is poor, sample sizes are small, and the risk of bias and imprecision is significant, necessitating the performance of more randomized controlled trials. Second, the type of inflammatory change, illness severity, microbiota features, and potential confounding factors, such as age, sex, food, and individual microbiological characteristics, must all be considered. Lastly, the anti-inflammatory efficacy of probiotics is incredibly reliant on the dose and strain. It is possible that different bacterial strains have different functions in relation to their hosts.

Due to limited survival rates and/or competition with the indigenous gut microbiome, delivering live bacteria *via* probiotics is difficult. Probiotic effects can be mediated *via* their metabolites or biological components, such as postbiotics. Amino acid derivatives altered by the gut microbiota could be a type of postbiotic that has anti-inflammatory properties by attaching to specific receptors on intestinal epithelial cells (Żółkiewicz et al., 2020). Because of their stability, synbiotics, such as “nonviable” microbial cells or crude cell extracts, can benefit both humans and animals if present in sufficient amounts (Vallejo-Cordoba et al., 2020). Probiotic bacteria can be manipulated in an unlimited manner with emerging biological engineering tools. In response to externally supplied substrates, genetically modified bacterial/probiotic strains can be employed to detect early inflammatory markers as well as to distribute and generate therapeutic compounds to the mucosal surface, hence boosting the overall efficiency of the system. It is also worthy of in-depth investigation for other gut microbiota-targeted adjuvant therapies of SpA, such as fecal microbiota transplantation (FMT) and dietary treatment.

## Some thoughts

1. It is time to rethink the heterogeneity of the SpA disease class and its underlying mechanisms. We only have a rudimentary understanding of the known associations with the microbiota in SpA-related diseases right now. However, the exact mechanisms through which the microbiome contributes to SpA in the axial or peripheral skeleton are still unknown, and the mechanisms by which the combined effects of the gut microbiome, immune system, and host genetics contribute to tissue damage will continue to be a hot topic for future research.
2. There is both commonality and specificity in dysbiosis among key disease subtypes, such as AS, IBD, and PsA, in SpA, implying a complicated relationship in the etiology of these heterogeneous disorders. The genetic background of these diseases has a high degree of overlap, but the clinical manifestations are significantly heterogeneous. It is not clear whether there is a connection between gut microbiota and joint lesions and whether there are other factors involved, weaving a relatively complex network of infection, immunity, and injury. Using SpA as an example to identify microbes that influence human disease susceptibility and phenotype will be a considerable challenge.
3. Does gut flora research have the potential to aid us in treating SpA with precision? Early diagnosis, early intervention, early treatment, and effective management of SpA have remained unsolved challenges in clinical practice, often leading to delayed diagnosis and precise individualized treatment. Advances in detection technologies such as sequencing and multiomics approaches such as metabolomics have allowed us to delve deeper into the links between ecological dysregulation, bacterial metabolites, and disease development and will provide new insights into the unraveling of this diverse group of diseases. Further research into mechanisms such as the ‘gut-joint’ axis may allow physicians to characterize SpA and disease progression with specific biomarkers to aid early diagnosis and provide individualized and precise treatment. The gut microbiome’s plasticity has also prompted researchers to assess the viability of precision therapy based on the gut microbiome’s SpA and to design new targeted therapies.

## Conclusions

The gut microbiota has emerged as a major focus of investigation into the pathogenesis of SpA. Although definitive proof of causality is still lacking, dysbiosis is linked to the pathogenesis of HLA-B27-associated SpAs. Notably, there is an interaction between the gut flora and the efficacy of current conventional therapeutic agents, and detailed studies on the gut-joint axis suggest that more targeted bDMARDs are a promising therapeutic area. Large-scale longitudinal studies and cross-sectional clinical trials are required to investigate the microbiota as a potential biomarker and its role in the prevention or treatment of SpA. According to the available literature, probiotics are extensively researched for use in SpA as adjunctive therapy. Nevertheless, the high heterogeneity in study design due to the use of different strains, quantities, and timing of supplementation makes it difficult to conclude whether probiotics are effective at this time. Engineered microbes, however, could be a more promising topic. Future microbiomics investigations and new analytical tools, such as bioinformatics, will allow for the more extensive study of host-microbiota interactions, providing new insights into the pathophysiology of SpA and, ideally, translating into therapeutically useful therapies.

## Author contributions

XL, JC, XG, and JY conceived and drafted the study. JY screened abstracts, XL, JC, and XG collected all data. XL and JC drafted the manuscript. JY performed critical revisions of the manuscript. All authors have approved the final draft of the manuscript.

## Funding

This work was supported by grants from National Nature Science Foundation of China (31870747, 32070724), Tianjin Natural Science Foundation Project (20JCYBJC00470).

## Conflict of interest

The authors declare that the research was conducted in the absence of any commercial or financial relationships that could be construed as a potential conflict of interest.

## Publisher’s note

All claims expressed in this article are solely those of the authors and do not necessarily represent those of their affiliated organizations, or those of the publisher, the editors and the reviewers. Any product that may be evaluated in this article, or claim that may be made by its manufacturer, is not guaranteed or endorsed by the publisher.
